# Dynamics of *Thioalkalivibrio* species in a co-culture under selective pressure of ampicillin

**DOI:** 10.1093/femsmc/xtad020

**Published:** 2023-11-08

**Authors:** Anne-Catherine Ahn, J Merijn Schuurmans, Dimitry Sorokin, Gerard Muyzer

**Affiliations:** Microbial Systems Ecology, Department of Freshwater and Marine Ecology, Institute for Biodiversity and Ecosystem Dynamics, University of Amsterdam, 1098 XH, Amsterdam, The Netherlands; Microbial Systems Ecology, Department of Freshwater and Marine Ecology, Institute for Biodiversity and Ecosystem Dynamics, University of Amsterdam, 1098 XH, Amsterdam, The Netherlands; Winogradsky Institute of Microbiology, Federal Research Centre of Biotechnology, Russian Academy of Sciences, 119071, Moscow, Russia; Department of Biotechnology, Delft University of Technology, 2629 HZ, Delft, The Netherlands; Microbial Systems Ecology, Department of Freshwater and Marine Ecology, Institute for Biodiversity and Ecosystem Dynamics, University of Amsterdam, 1098 XH, Amsterdam, The Netherlands

**Keywords:** antibiotics, haloalkaliphilic, population dynamics, soda lakes, sulfur-oxidizing bacteria, *Thioalkalivibrio*

## Abstract

Haloalkaliphilic chemolithoautotrophic sulfur-oxidizing bacteria belonging to the genus *Thioalkalivibrio* are highly abundant in microbial communities found in soda lakes and dominant in full-scale bioreactors removing sulfide from industrial waste gases. Despite certain soda lakes being remote and unaffected by anthropogenic activities, haloalkaliphilic microorganisms, including *Thioalkalivibrio* strains, possess various antibiotic resistance genes. In this study, we investigated the impact of the antibiotic ampicillin on a co-culture of two *Thioalkalivibrio* species*, Tv. thiocyanoxidans* ARh2^T^ and *Tv. versutus* AL2^T^, both experimentally and through *in silico* analysis of antibiotic resistance. Cell growth dynamics were monitored over time at increasing ampicillin concentrations using rep- and qPCR. Within ten days after the addition of ampicillin, the co-culture transitioned from a *Tv. thiocyanoxidans* ARh2^T^-dominated to a stable *Tv. versutus* AL2^T^-dominated culture. This shift was attributed to *Tv. versutus* AL2^T^ displaying a lower susceptibility to ampicillin, making it more competitive. These results emphasize the potential implications of antibiotic pressure on microbial communities, where a resistant species can outcompete a stable co-culture. This study presents the first evidence of such dynamics in haloalkaliphilic chemolithoautotrophs. By understanding the antibiotic resistance and the competitive dynamics of haloalkaliphilic bacteria like *Thioalkalivibrio*, we can gain insights into their behaviour and stress response.

## Introduction

Soda lakes are unique stable haloalkaline environments harbouring diverse microbial communities dominated by prokaryotes (Sorokin et al. 2014, Sorokin et al. 2015). These lakes can be found worldwide in areas with arid climates and with groundwater rich in sodium bicarbonate. High evaporation rates transform these lakes into highly sodium carbonate/bicarbonate-buffered, stable alkaline systems able to reach salt saturation (Jones et al. 1977, Grant et al. 1990, Jones et al. 1998). Despite these extreme conditions, the microbial communities are well adapted and flourish in these environments (Jones et al. 1998, Ochsenreiter et al. 2002, Mesbah et al. 2007, Lanzén et al. 2013), in which the chemolithoautotrophic sulfur-oxidizing bacteria of the genus *Thioalkalivibrio* are abundantly present (Sorokin et al. 2013, Vavourakis et al. 2016, Edwardson and Hollibaugh 2017, 2018, Zorz et al. 2019). In addition to soda lakes, *Thioalkalivibrio* strains are also important members of the microbial communities in the so-called “Thiopaq process”, in which they sustainably remove sulfide from industrial waste gas through oxidation to elemental sulfur under haloalkaliphilic conditions (Sorokin et al. 2008, 2012). Recently, the antibiotic and metal “resistome” of the microbial communities of Lonar Lake, an anthropogenic-polluted alkaline lake in India, was described (Chakraborty et al. 2020a,b), for which *Thioalkalivibrio* has been significantly associated with antibiotic resistance genes (Chakraborty et al. 2020a).

The increased occurrence of antibiotic resistant bacteria and their associated genes in waterbodies due to anthropogenic pressure is an alarming phenomenon (Chakraborty et al. 2020b, Zhang et al. 2009, Vaz-Moreira et al. 2014, Yang et al. 2017, Su et al. 2020). However, antibiotic resistance as result of long-term evolution, existed long before anthropogenic pressure (D´Costa et al. 2011, Perry et al. 2016), protecting bacteria against their own antibiotic production and enabling them to outcompete other microbes in the ecosystem (Fajardo et al. 2009, Granato et al. 2019). Applying sub-inhibitory concentrations in humans, livestock or the environment poses a major threat to bacterial disease treatments with antibiotics, as it leads to an increase of antibiotic resistance amongst pathogens (Andersson and Hughes 2012). Alternatively, an antibiotic susceptible strain can be swept out of the population by an emerging resistant strain, which initially composed only a small fraction of the population, if an antibiotic concentration between the minimal inhibitory concentration (MIC) of the susceptible strain and the MIC of the resistant strain is applied. This concept is described as the “mutant selection window” hypothesis (Negri et al. 1994, 2000, Drlica and Zhao 2007).

Even though certain soda lakes are situated in remote regions without anthropogenic pressure, various haloalkaliphilic microorganisms from those environments possess a diverse spectrum of different antibiotic resistances (Pikuta et al. 2003, Boldareva et al. 2008, 2009a,b, Wu et al. 2010, Zargar et al. 2010, Kompantseva et al. 2012, Liu et al. 2012, Kiplimo et al. 2019). This has also been shown for strains of *Thioalkalivibrio* (Chakraborty et al. 2020a). With the interest to explore the antibiotic resistance capacity within *Thioalkalivibrio*, we studied here the dynamics of two *Thioalkalivibrio* species growing as a co-culture at increasing concentrations of ampicillin over a period of four weeks followed by four weeks without antibiotics. The dynamics in the co-culture were monitored with repetitive-element PCR (rep-PCR), which is a molecular fingerprint technique able to distinguish between the two *Thioalkalivibrio* species, and with quantitative PCR (qPCR) targeting strain-specific genes.

## Material and methods

### Cultivation and adaptation

Cultures of *Tv. thiocyanoxidans* ARh2^T^ with low numbers of *Tv. versutus* AL2^T^ were grown in 10 ml batch cultures at 100 rpm and 30°C with increasing concentrations of ampicillin for four weeks followed by four weeks without the addition of ampicillin. During the ampicillin adaptation phase, the culture was changed at each transfer to fresh medium with a doubled concentration in ampicillin, but it was also transferred again to fresh medium with the same ampicillin concentration. This was in case the culture would not be able to grow at the increased ampicillin concentration and needed more time to adapt at the lower concentration. The cultures started at 0.1 µg/ml ampicillin and were able to grow until 12.8 µg/ml. No growth could be obtained with 25.6 µg/ml. The experimental transfer schedule of the complete experiment can be found in Table S1 and is illustrated for the adaptation phase in Fig. S1. For each new culture, a 1:200 dilution of the previous culture was applied during the serial transfers. The medium was composed of 17.5 g/l Na_2_CO_3_, 13.9 g/l NaHCO_3_, 6.1 g/l NaCl, 1 g/l K_2_HPO_4_, 0.2 g/l MgCl_2_, 40 mM Na_2_S_2_O_3_, 5 mM NH_4_Cl and 1:1 000 trace metals (Pfennig and Lippert 1966), and the pH was adjusted to pH 9.8. Before inoculating the bacteria, a sterile solution of ampicillin (Roche Diagnostics GMBH, Mannheim, Germany) was added to the medium. Cultures with added ampicillin were prepared in triplicates and one culture without ampicillin was used as a reference. The MIC of both species was tested in triplicate using the same culture medium and the same ampicillin concentrations as for the adaptation experiment. Growth of the bacteria was followed daily by measuring the optical density in triplicates at 600 nm, and the average and the standard deviation of the three measurements were used in the data analysis.

### Repetitive-element PCR

Samples were taken in the exponential phase at the end of each adaptation step and were pelleted by centrifugation at 15 000 × *g* for 2 min. The supernatant was removed and the pellets were stored at −20°C until further processing. Genomic DNA was extracted with the DNeasy Blood & Tissue Kit (Qiagen, Venlo, The Netherlands) following the supplier's instructions for Gram-negative bacteria.

Rep‐PCR genomic fingerprinting was performed using the BOX A1R (5′‐CTACGGCAAGGCGACGCTGACG‐3′) primer (Versalovic et al. 1994). The PCR protocol included an initial denaturation step at 94°C for 3 min, followed by 30 cycles of two denaturation steps of 94°C for 3 sec and 92°C for 3 sec, an annealing step of 46.4°C for 1 min, and an elongation step at 72°C for 8 min, and a final elongation step at 72°C for 8 min (Foti et al. 2006). The reaction included in a total reaction volume of 25 µl, the Taq PCR Master Mix Kit 2x (Qiagen, Venlo, The Netherlands), 0.5 µM primer, and 90 ng of template DNA. Rep-PCR products were visualized on a 1.5% (w/v) agarose gel stained with 0.6 µg/ml ethidium bromide, which ran at 60 V for 8 h at 4°C. The GeneRuler DNA Ladder Mix (Thermo Fisher Scientific, Waltham, MA, USA) was used as a marker.

### Quantitative PCR

The qPCR was performed in an ABI 7500 Real-Time PCR system (Applied Biosystems, Waltham, MA, USA) with strain-specific primers on the same template DNA as were used for the rep-PCR. The primers were designed with the Primer3 program (https://primer3.ut.ee) and are listed with their target strain and genes in Table 1. The specificity of the primers was assessed *in silico* by BLAST and confirmed by a PCR on genomic DNA of *Tv. versutus* AL2^T^, *Tv. thiocyanoxidans* ARh2^T^ and a 1:1 mixture of both species. The PCR protocol included an initial denaturation step at 94°C for 3 min, followed by 35 cycles of a denaturation at 94°C for 30 sec, an annealing at 60°C for 30 sec and an extension at 72°C for 15 sec, and a final extension at 72°C for 10 min in 25 µl of reaction mix containing Taq PCR Master Mix Kit 2x (Qiagen, Venlo, The Netherlands), 0.5 µM primer, and 10 ng DNA. PCR products were revealed by gel electrophoresis (Figure S2). The qPCR protocol included an initial denaturation at 95°C for 10 min, followed by 40 cycles of a denaturation at 95°C for 15 sec, an annealing at 60°C for 30 sec and an extension at 72°C for 30 sec. The reaction mix contained Maxima SYBR Green qPCR Master Mix (2x) (Thermo Fisher Scientific, Waltham, MA, USA), 0.3 µM of forward and reverse primers, and 2 ng DNA in a total reaction volume of 25 µl. ROX solution (Thermo Fisher Scientific, Waltham, MA, USA) was used to correct for well-to-well variation and melting curves were analysed for all measured samples to exclude non-specific PCR products. Each qPCR plate contained a reference sample (0 µg/ml-Ref) and a reference gene (16S rRNA) to overcome plate effects. For each individual sample, three technical replicates were measured, and the average and the standard error of the three measurements were used in the data analysis.

**Table 1.** Primers used in the qPCR.

Gene name*	Sequence (5´-3´) (Forward or Reverse)	Target strain (Locus tag)	Fragment length (bp)
16S rRNA	CGTGTGTGAAGAAGGCCTG (F)	AL2^T^ (B0684_RS11480), ARh2^T^ (G372_RS0102255)	104
	CCGGTGCTTCTTCTGTAGGT (R)		
TcDH	AATACGTACTGCTGGCACCG (F)	ARh2^T^ (G372_RS0106320)	96
	AGTTCCATCCAGCCAACCAC (R)		
PMOR	GTGCGTATCCTCTTCGACC (F)	AL2^T^ (B0684_RS04235)	102
	ATCACAGAACTGCCCGTGG (R)		

* TcDH encodes the thiocyanate: cytochrome c oxidoreductase; PMOR encodes the peptide-methionine (S)-S-oxide reductase.

Individual amplification efficiencies (E) were calculated with LinRegPCR v.2018.0 (Ramakers et al. 2003, Ruijter et al. 2009) and were found between 1.8 and 2.0 with the exception at very low target numbers. The data was baseline corrected and threshold cycle (C_t_) values were calculated using LinRegPCR. Relative gene quantification was calculated using the initial culture before adaptation (T_0_) as reference sample and the 16S rRNA as reference gene according to the 2^−ΔΔCt^ method (Livak and Schmittgen 2001). This type of quantification compares the changes in the target gene´s copy number in a sample of interest to those in a reference sample (Livak and Schmittgen 2001). Negative control samples, in which the DNA template was replaced by water, did not show amplification.

### *In silico* analysis on ampicillin resistance genes

The genomes of *Tv. versutus* AL2^T^ (NZ_MVAR00000000) and *Tv. thiocyanoxidans* ARh2^T^ (NZ_ARQK00000000) were previously sequenced and annotated. The gene sequences of both species were obtained from the NCBI RefSeq FTP server. Blastp analyses were performed to detect the presence of beta-lactamase genes in the genome of *Tv. versutus* AL2^T^ and *Tv. thiocyanoxidans* ARh2*^T^*. For this, the beta-lactamase protein gene sequences of TEM, SHV, OXA and AmpC from other Gammaproteobacteria were used as references (Uniprot: Q6SJ61, Q0QXE5, Q5MD99, P0A9Z8, C7S9D0, P0A3M3, Q5EET9, D6NSM8). Moreover, the KEGG pathway map for beta-lactam resistance was analyzed for both strains and genes encoding for multidrug efflux pumps, RND family efflux transporters, AcrAB-TolC efflux pumps and MFS transporters were retrieved for analysis from the genomes of both species.

## Results

### Revealing ampicillin-driven species shifts in the co-culture

**Figure 1.**
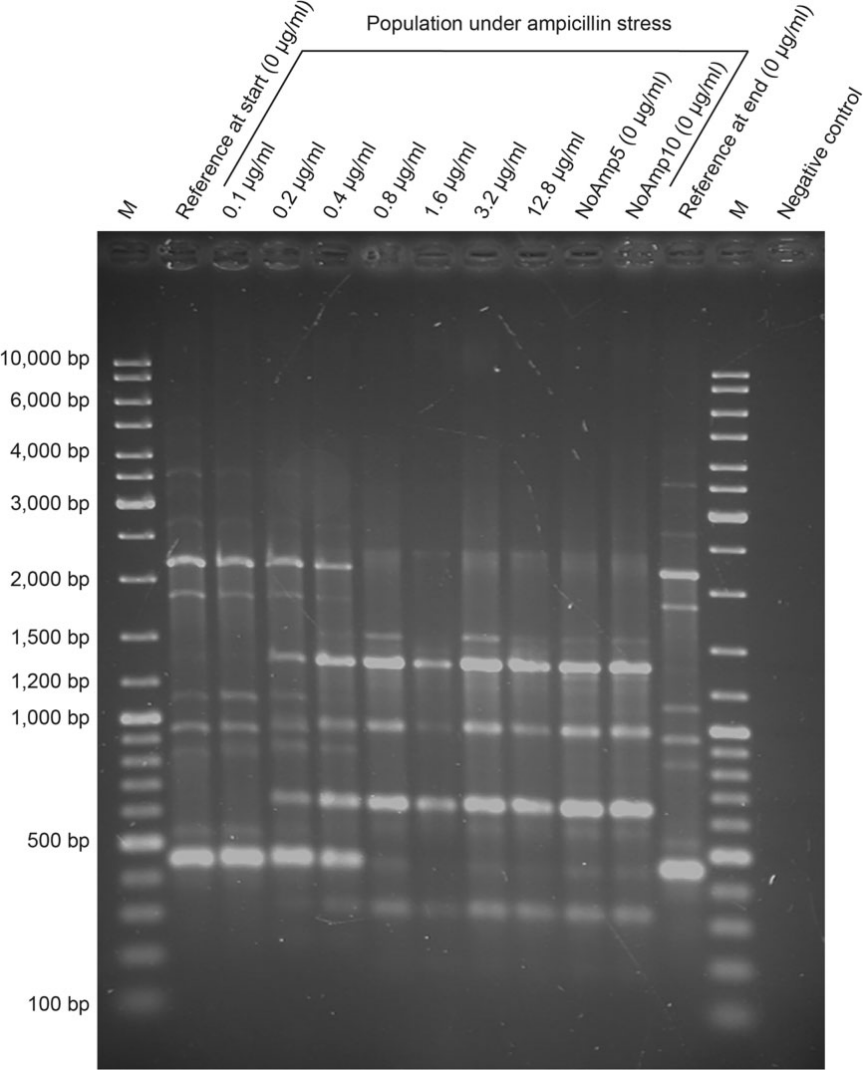
Rep-PCR patterns showing the dynamics of a co-culture of *Tv. thiocyanoxidans* ARh2^T^ and *Tv. versutus* AL2^T^ incubated with increasing ampicillin concentrations over time. The reference is a culture from the same starting culture as the adapted cultures, but without addition of ampicillin. NoAmp5 and NoAmp10 represent the adapted cultures after five and ten transfers without ampicillin, respectively. M represents the DNA marker.

The rep-PCR patterns of a culture treated with increasing ampicillin concentrations and of the reference grown in the absence of ampicillin were followed in time (Fig. 1). The rep-PCR pattern of *Tv. thiocyanoxidans* ARh2^T^ was present for the initial culture and for the culture containing 0.1 µg/ml ampicillin. A mix of both species’ rep-PCR fingerprint was seen for the culture at 0.2 µg/ml and at 0.4 µg/ml. The rep-PCR fingerprint then switched completely to the fingerprint of *Tv. versutus* AL2^T^ at ampicillin concentrations of 0.8 µg/ml and higher. However, the reference culture incubated without ampicillin, did not experience a shift in pattern and maintained its *Tv. thiocyanoxidans* ARh2^T^ pattern. Characteristic bands with the BOX A1R primer were found for *Tv. thiocyanoxidans* ARh2^T^ at ∼470 bp, ∼520 bp, ∼850 bp, ∼950 bp, ∼1100 bp, ∼1850 bp and ∼2200 bp, whereas for *Tv. versutus* AL2^T^ they were found at ∼320 bp, ∼510 bp, ∼650 bp, ∼960 bp, ∼1300 bp, ∼1550 bp, and ∼2300 bp.

**Figure 2.**
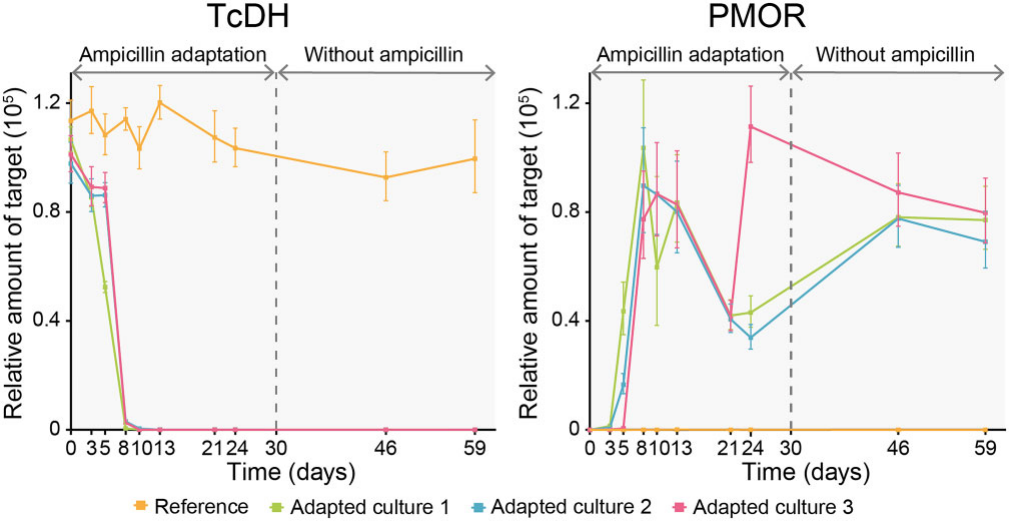
Dynamics of the co-culture followed over time by qPCR with TcDH, which is specific to *Tv. thiocyanoxidans* ARh2^T^ and PMOR, which is specific to *Tv. versutus* AL2^T^. Adapted culture 1, 2, and 3 represent triplicates from the same starting culture and were kept separate from each other throughout the experiment. The reference is a culture from the same starting culture as the adapted cultures, but without addition of ampicillin. The error bars depict the standard error of the average.

The relative abundance of *Tv. thiocyanoxidans* ARh2^T^ and *Tv. versutus* AL2^T^ in the culture was monitored using qPCR amplification of strain-specific genes (Fig. 2). The gene encoding for thiocyanate: cytochrome *c* oxidoreductase (TcDH) was selected for *Tv. thiocyanoxidans* ARh2^T^ and the gene encoding the peptide-methionine (S)-S-oxide reductase (PMOR) for *Tv. versutus* AL2^T^ as they are single-copy genes and not present in the respective other species. The 16S rRNA gene is also only present as a single-copy gene in both species and was used as a reference for normalisation. The specificity of all three primers was evaluated as good after testing them *in silico* as well as by PCR using the genomic DNA of both species and a 1:1 mixture of DNA of both species (Figure S2).

The reference culture, which was incubated without ampicillin, maintained its initial composition of *Tv. thiocyanoxidans* ARh2^T^ in the qPCR. However, cultures with increasing ampicillin concentration started to shift their composition from a *Tv. thiocyanoxidans* ARh2^T^-dominated culture as seen with 0 and 0.1 µg/ml ampicillin to a mixture of *Tv. thiocyanoxidans* ARh2^T^ and *Tv. versutus* AL2^T^ when the ampicillin concentration increased to 0.2 and 0.4 µg/ml between day three and eight. The original species *Tv. thiocyanoxidans* ARh2^T^ was fully outcompeted by *Tv. versutus* AL2^T^ after an increase to 0.8 µg/ml ampicillin sampled at day 10. The initial composition with *Tv. thiocyanoxidans* ARh2^T^ did not recover after ten successive incubations without ampicillin over a period of four weeks.

### Sensitivity of pure cultures to ampicillin

To understand the shift in species composition after incubation with the increasing concentrations of ampicillin, the sensitivity of *Tv. versutus* AL2^T^ and *Tv. thiocyanoxidans* ARh2^T^ was tested using the same concentrations as in the adaptation experiment (Figure S3). *Tv. versutus* AL2^T^ was able to grow with 0.1 µg/ml after two days and with 0.2 and 0.4 µg/ml after three days lag period whereas *Tv. thiocyanoxidans* ARh2^T^ showed only limited growth with ampicillin with 0.1 µg/ml after only three days. From a concentration of 0.8 µg/ml and of 0.2 µg/ml onwards, *Tv. versutus* AL2^T^ and *Tv. thiocyanoxidans* ARh2^T^, respectively, were only able to grow after four days of incubation, which is likely due to degradation of ampicillin in the growth medium. Therefore, the respective MIC for ampicillin for *Tv. versutus* AL2^T^ and *Tv. thiocyanoxidans* ARh2^T^ are 0.8 µg/ml and 0.2 µg/ml. Chemical degradation of ampicillin in the culture medium was tested by inoculation of *Thioalkalivibrio* after one, two, three, or four days. Independently of when *Thioalkalivibrio* was added, visible growth only started after the fourth day when ampicillin was added to the medium (data not shown). In the absence of ampicillin, both species showed growth within one day. The OD_600_ peak at the start of each *Thioalkalivibrio* culture resulted through the presence of elemental sulfur produced by the oxidation of thiosulfate. Its decrease after one day is linked to the depletion of sulfur due to complete oxidation to sulfate.

### Genomic exploration of ampicillin resistance genes in *Thioalkalivibrio*

Analysis of the genome sequences of *Tv. versutus* AL2^T^ and *Tv. thiocyanoxidans* ARh2*^T^* was performed to retrieve their ampicillin and general antibiotic resistance capacity and to reveal differences. No beta-lactamase protein gene sequences of TEM, SHV, OXA and AmpC could be detected in either of the *Thioalkalivibrio* genomes using BLASTp against known protein sequences of other Gammaproteobacteria. The KEGG pathway map for beta-lactam resistance showed limited presence of genes in both species, but included penicillin-binding proteins and multidrug efflux pumps and *Tv. versutus* AL2^T^ only possesses one supplementary gene (B0684_RS10505), which could not be found in *Tv. thiocyanoxidans* ARh2^T^ (Figure S4, Figure S5, Table S2). Both genomes were also screened for genes annotated as putative multidrug efflux pumps, putative RND family efflux transporters, putative AcrAB-TolC efflux pumps and putative MFS transporters (Table S2). Most genes could be retrieved in both genomes, but four genes are unique to *Tv. thiocyanoxidans* ARh2^T^, whereas eight to *Tv. versutus* AL2^T^. For *Tv. thiocyanoxidans* ARh2^T^, those genes are a multiple antibiotic resistance protein MarC (G372_RS0106695), multidrug resistance efflux pump (G372_RS0103685), multidrug efflux pump subunit AcrB (G372_RS0103690) and an outer membrane protein TolC (G372_RS0109555). For *Tv. versutus* AL2^T^, those include two multidrug efflux pump subunits AcrB (B0684_RS03445, B0684_RS12195), two RND family efflux transporter-MFP subunits (B0684_RS03450, B0684_RS09870), an outer membrane protein TolC (B0684_RS04965), a multiple antibiotic resistance protein MarC (B0684_RS07955), an outer membrane protein-multidrug efflux system (B0684_RS08515), and a multidrug resistance efflux pump (B0684_RS12200).

## Discussion

Our study supports the concept of the “mutant selection window” hypothesis. This concept states that a resistant subpopulation, which is initially only present as a minor fraction in the population, can emerge and outcompete a susceptible subpopulation from the population, if an antibiotic concentration between the MIC of the susceptible and the MIC of the resistant subpopulation is applied (Negri et al. 1994, 2000, Drlica and Zhao 2007). To prevent emergence of resistance from a pre-existing subpopulation, a “mutant prevention concentration” needs to be applied, which is a concentration higher than the MIC of the most resistant subpopulation (Drlica and Zhao 2007). This phenomenon is described based on two mutant subpopulations of a strain. However, the same principle can also be applied on two strains of a species, or on two species of a genus with the latter being the case in our study. The “mutant selection window” hypothesis was relativised by the study of Alexander and Maclean (2020). They predicted that at one-eighth of their MIC, individual resistant cells have only a less than 5% probability of detectable outgrowth. Moreover, they suggest that antibiotic concentrations below the MIC of the resistant strain might be efficient enough to eradicate the bacterial population when resistant mutants only make up a small fraction in the beginning (Alexander and Maclean 2020). However, it has been shown in our study as well as in multiple others that the selective antibiotic pressure can impact the population composition when a concentration below the MIC of the sensitive strain was applied. This provides a window of opportunity for resistant strains to outcompete the susceptible ones (Negri et al. 2000, Gullberg et al. 2011, Andersson and Hughes 2012, Hughes and Andersson 2012, Day et al. 2015).

Screening the composition of the co-culture with rep- and qPCR during the incubation with increasing concentrations of ampicillin revealed a switch in species dominance of the co-culture. Notably, the more resistant species *Tv. versutus* AL2^T^, which in the beginning of the experiment was only a minor component of the co-culture and not detectable by qPCR, outcompeted the more susceptible species *Tv. thiocyanoxidans* ARh2^T^. Within ten days and with an eight-fold increase of the ampicillin concentration, the resistant species became dominant in the co-culture and the sensitive species was no longer detectable. Growth profiles with different ampicillin concentrations explained this shift in dominance by a higher MIC for *Tv. versutus* AL2^T^. Competitivity of *Tv. thiocyanoxidans* ARh2^T^ in the absence of ampicillin pressure as seen in the control culture could be explained on the other hand by the theory that the resistant strain has a higher fitness cost, for which reason the sensitive strain can outcompete the resistant strain (Gullberg et al. 2011).

The *in silico* analysis of both species´ genomes did not detect any beta-lactamase genes and only revealed a limited number of genes associated with beta-lactam resistance. However, multiple putative multidrug efflux pump, putative RND family efflux transporters, putative AcrAB-TolC efflux pumps and putative MFS transporters, which have been previously associated to antibiotic resistance (Nikaido and Takatsuka 2009, Pérez et al. 2012, Nag and Mehra 2021), could be detected in both genomes with four genes specific to *Tv. thiocyanoxidans* ARh2^T^ and eight to *Tv. versutus* AL2^T^. It is possible that amongst the specific genes in *Tv. versutus* AL2^T^, a gene could be present allowing its increase resistance to ampicillin compared to *Tv. thiocyanoxidans* ARh2^T^. However, their specific function and implication in ampicillin resistance still remains to be proven experimentally. Supplementary transcriptomic and proteomic analysis are needed to gain insights in the molecular mechanisms of ampicillin resistance in *Thioalkalivibrio* and to gain a better understanding of the increased ampicillin resistance of *Tv. versutus* AL2^T^.

Antibiotic treatments are associated with the evolution and the spread of resistances within microorganisms (Blair et al. 2014), as well as with shifts within the microbial community composition. These dynamics include changes in abundance and extinction of species in a given community (Coates et al. 2018, Cairns et al. 2020). Already a population composed by a single strain and subjected to bactericidal antibiotics showed population dynamics, but no stochastic dynamic was observed with bacteriostatic antibiotics (Coates et al. 2018). In a recent study on the impact of antibiotic perturbations on a complex microbial consortium, Cairns et al. (2020) found a repeatable community response. In their experiment, mock communities composed of 34 bacterial species were challenged with a pulsed antibiotic disturbance at different concentrations, with or without species immigration, to study the ecological community response. They proposed that this response could be linked to the antibiotic susceptibility and the growth rate of each given species. Next to species sorting, also antibiotic resistance evolution occurred in their experiment and its magnitude increased with increasing concentration of antibiotics. However, the genomic evolution seemed not to be relevant for the short-term ecological dynamics in the studied community as the experiment showed high repeatability amongst the replicates (Cairns et al. 2020). In our study, a growing culture could not be obtained with ampicillin concentration higher than 12.5 µg/ml, which suggests a lack of rescuing genetic mutations in the final *Tv. versutus* AL2^T^-dominated co-culture.

Our results demonstrate how a stable population, which is dominated by a susceptible species can be exchanged by a stable population composed by a resistant species due to antibiotic pressure. To our knowledge it is the first time that the application of the “mutant application window” hypothesis is shown for haloalkaliphilic chemolithoautotrophs.

## Supplementary Material

xtad020_Supplemental_Files
