# Supplementary material for: Dynamics of Thioalkalivibrio species in a co-culture under selective pressure of ampicillin
Source: FEMS Microbes. 2023 Nov 8;4:xtad020. doi: 10.1093/femsmc/xtad020 (PMC10699867; doi:10.1093/femsmc/xtad020)
Supplement: xtad020_Supplemental_Files [file xtad020_supplemental_files.zip › Supplementary File.docx]

Supplementary Material

Dynamics of *Thioalkalivibrio* species in a co-culture under selective pressure of ampicillin

Anne-Catherine Ahn^1^, J. Merijn Schuurmans^1^, Dimitry Sorokin^2,3^, Gerard Muyzer^1^

^1^ Microbial Systems Ecology, Department of Freshwater and Marine Ecology, Institute for Biodiversity and Ecosystem Dynamics, University of Amsterdam, Amsterdam, The Netherlands

^2^ Winogradsky Institute of Microbiology, Federal Research Centre of Biotechnology, Russian Academy of Sciences, Moscow, Russia

^3^ Department of Biotechnology, Delft University of Technology, Delft, The Netherlands


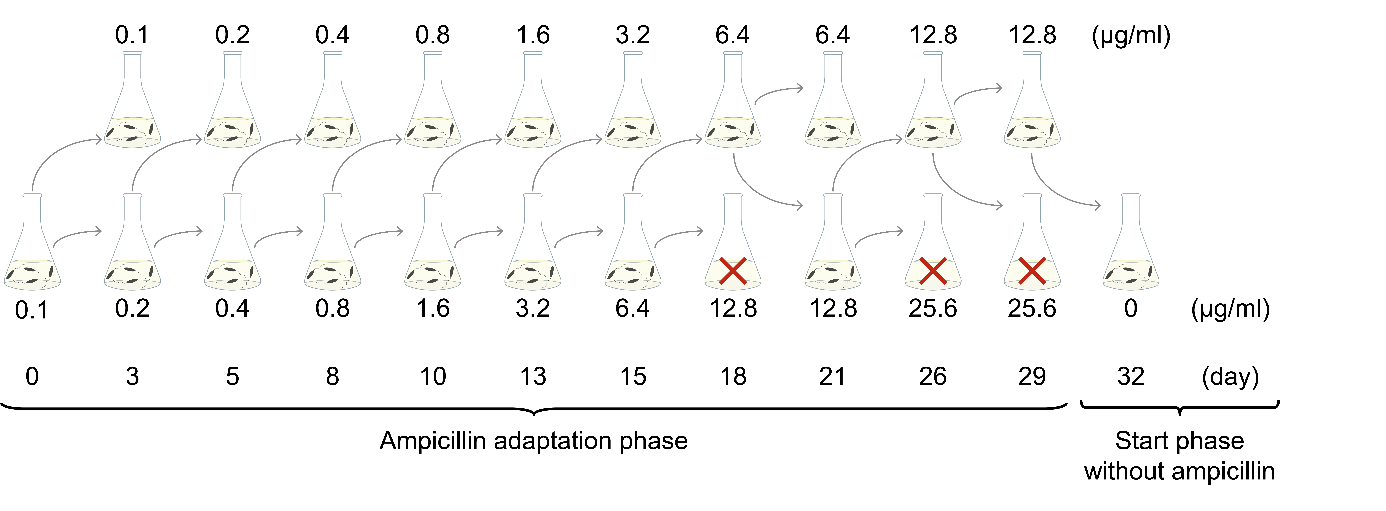


**Figure S1. Experimental transfer schedule for the ampicillin adaptation phase with ampicillin concentrations and transfer times.** Cultures with bacteria pictured in Erlenmeyer showed growth whereas cultures with red crosses did not show growth.


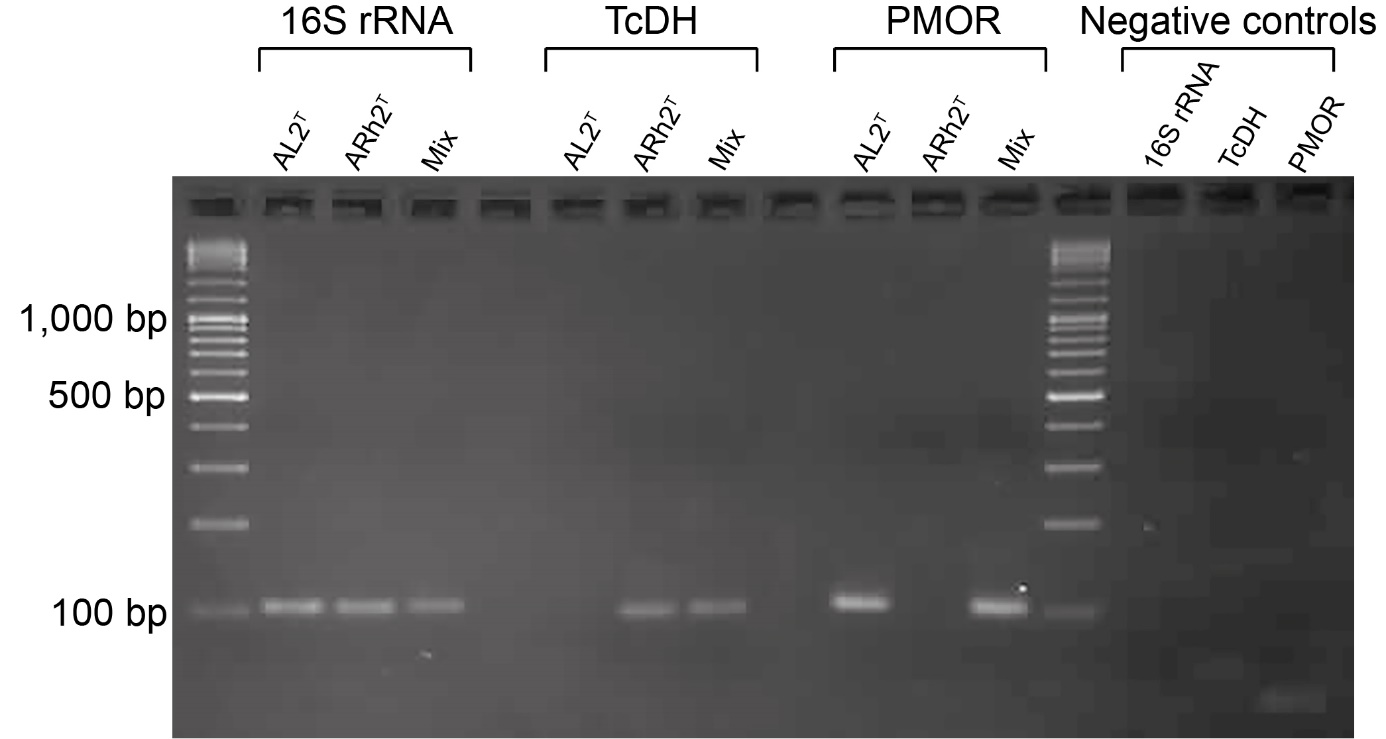


**Figure S2. Primer specificity of 16S rRNA, TcDH and PMOR.** The agarose gel shows that 16S rRNA amplifies both strains, whereas TcDH is specific for *Tv. thiocyanoxidans* ARh2^T^ and PMOR for *Tv. versutus* AL2^T^. All primers were able to amplify in the 1:1 mix.


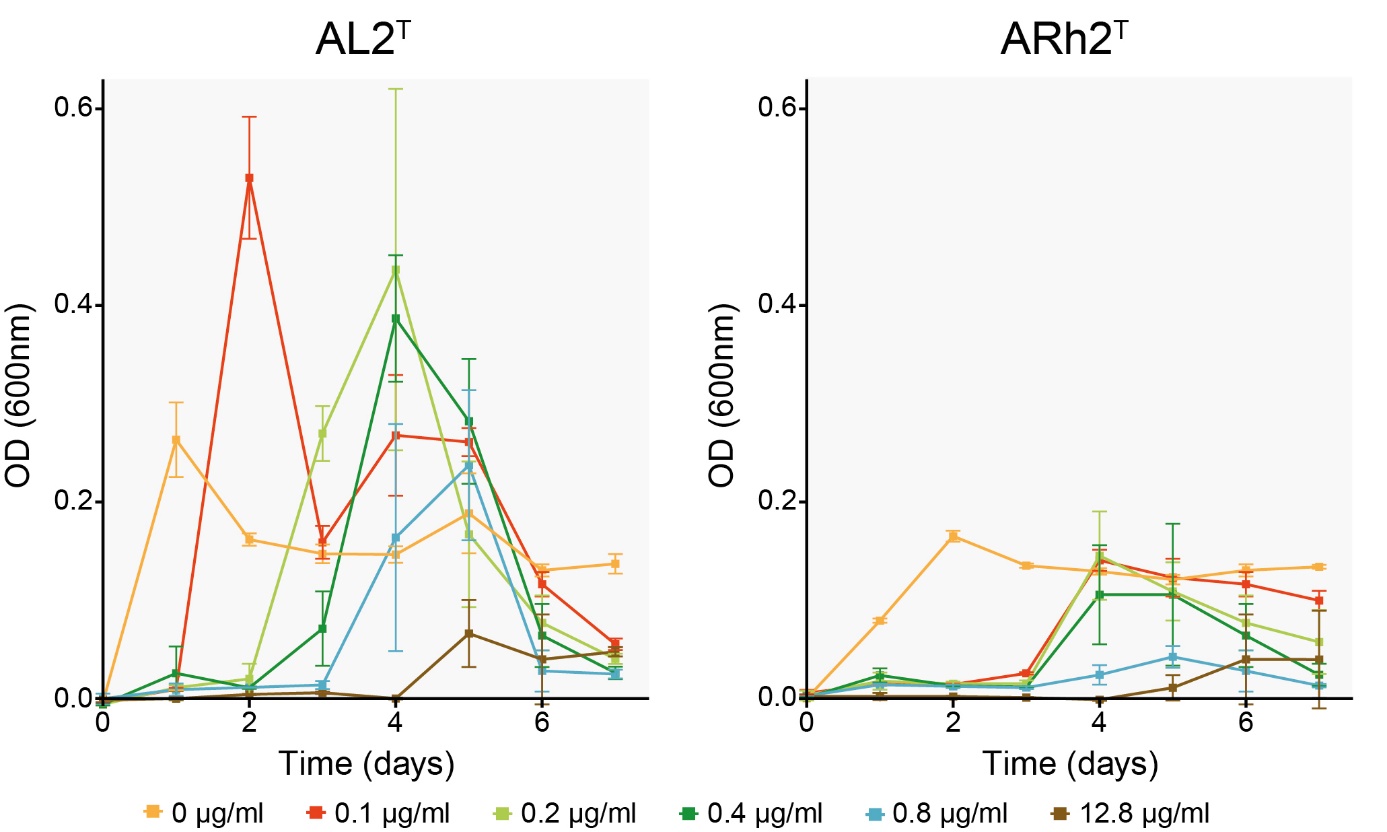


**Figure S3. Antibiotic sensitivity of *Tv. thiocyanoxidans* ARh2^T^ and *Tv. versutus* AL2^T^ to different ampicillin concentrations.** The error bars depict the standard deviation of the average.


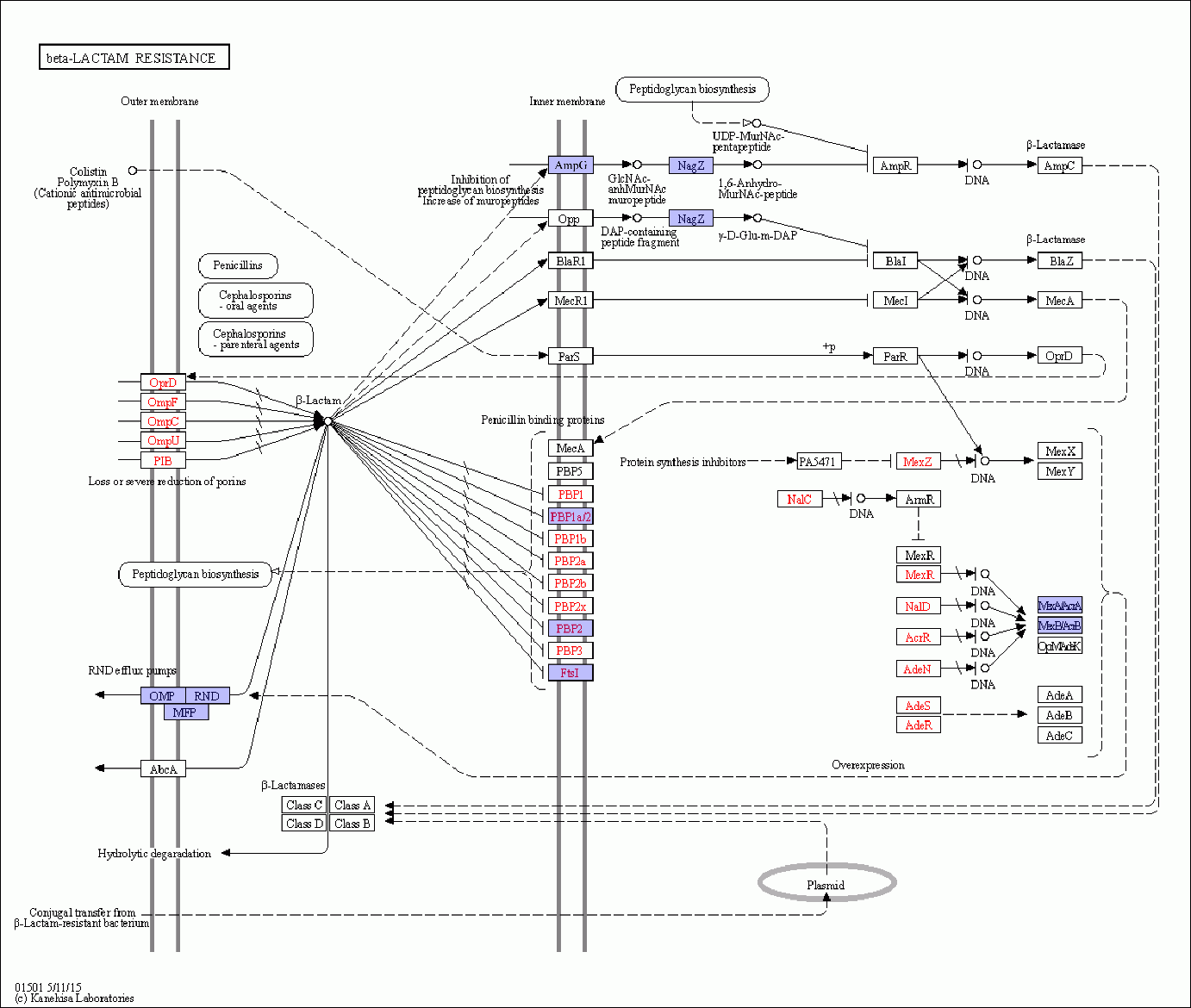


**Figure S4. KEGG pathway map of encoded beta-lactam resistance genes in the genome of Tv. versutus AL2^T^.** The KEGG pathway map represents genes, which have been associated with beta-lactam resistance, but only the genes in cases coloured in violet are present in the genome of Tv. versutus AL2^T^. Genes with names in red have been associated in literature with pathogenicity.


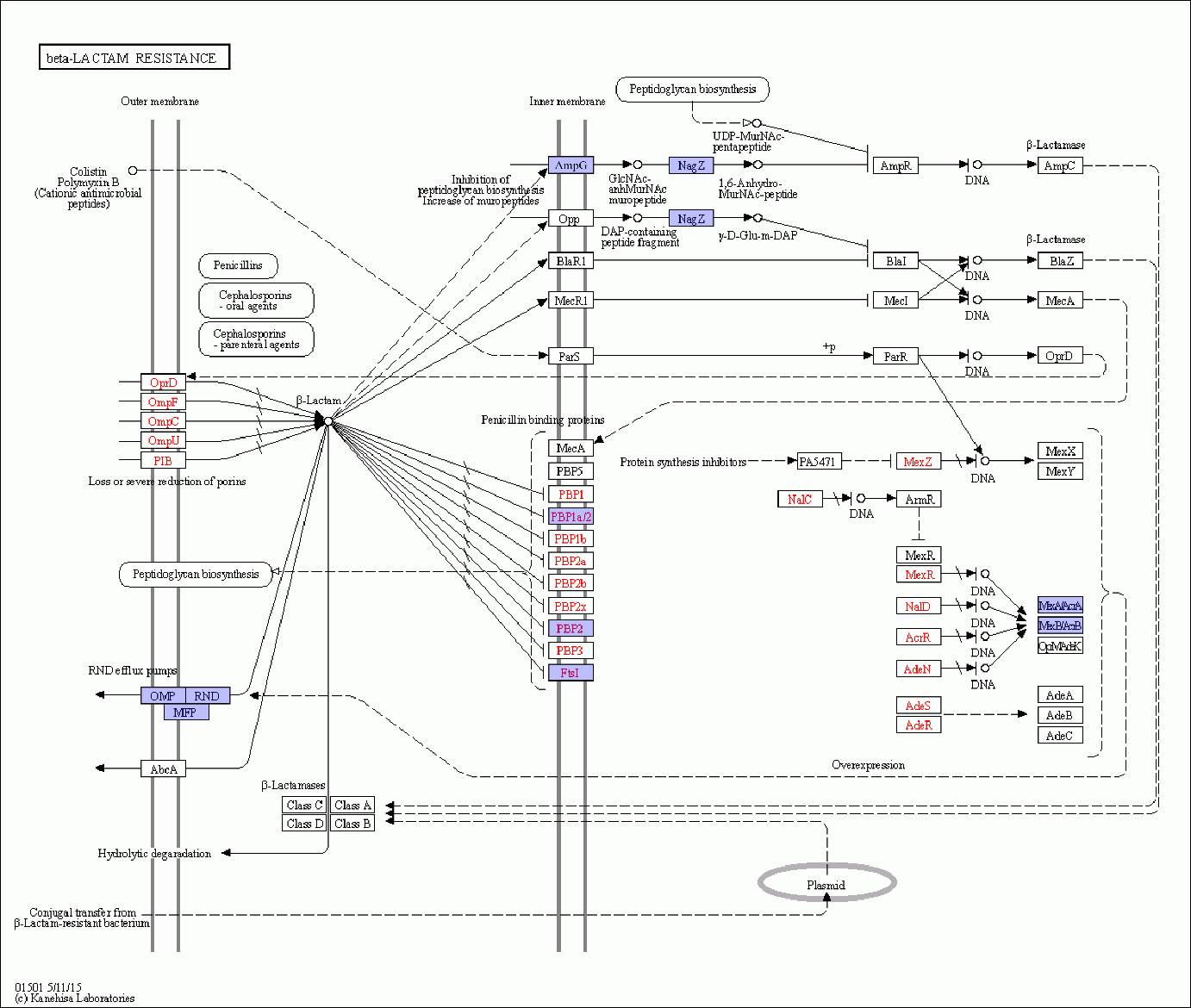


**Figure S5. KEGG pathway map of encoded beta-lactam resistance genes in the genome of Tv. thiocyanoxidans ARh2^T^.** The KEGG pathway map represents genes, which have been associated with beta-lactam resistance, but only the genes in cases coloured in violet are present in the genome of Tv. thiocyanoxidans ARh2^T^. Genes with names in red have been associated in literature with pathogenicity.

**Table S1. Experimental transfer schedule with the start and end of each culture in days and the respective ampicillin concentrations added.**

**Table S2. Genes implicated in the beta-lactam resistance found by the KEGG pathway map and genes annotated as putative multidrug efflux pumps, RND family efflux transporters, AcrAB-TolC efflux pumps and MFS transporters encoded in the genomes of Tv. versutus AL2^T^ and Tv. thiocyanoxidans ARh2^T^.**
