# Supplementary material for: Dynamics of Thioalkalivibrio species in a co-culture under selective pressure of ampicillin
Source: FEMS Microbes. 2023 Nov 8;4:xtad020. doi: 10.1093/femsmc/xtad020 (PMC10699867; doi:10.1093/femsmc/xtad020)
Supplement: xtad020_Supplemental_Files [file xtad020_supplemental_files.zip › FEMSMC-2023-004_supplementary_data_-_reviews_and_author_response.docx]

**Reviews of manuscript FEMSMC-2023-004**

**Reviewer 1: Horia Banciu, Babes-Bolyai University**

**Recommendation: Major Revision**

The manuscript (ID FEMSMC-2023-004) entitled ” Population dynamics of Thioalkalivibrio species under selective pressure of ampicillin” by Ahn et al. is a hypothesis-testing research dealing with a particular case of haloalkaliphilic community response to antibiotics.

I overall found the manuscript interesting and enjoyable to read. Authors used straightforward approaches to test antibiotic-driven changes in mixed cultures.

My concerns and suggestions are listed below:

1. The members of genus Thioalkalivibrio are now genome-sequences, therefore, I believe that addition of a short in silico analysis on the antibiotic determinants (eventually on those conferring beta-lactam resistance) would be of interest. I wonder whether the genome of AL2 strain encodes such genes. This analysis would be definitively prove insightful and may explain the experimental results.

2. In the Abstract and Introduction, authors should briefly reasoning on why they experimented on this certain physiological (haloalkaliphiles) and metabolical (sulfur-oxidizers) types of bacteria.

3. In addition to the above concern, the authors are kindly asked to explain why they used ampicillin (beta-lactam antibiotic) that is usually effective against Gram-positives as the tested strains are Gram negative.

4. The use of the term “population” instead of “mixed culture” is problematic especially due to the established definition of biological population that consists of a number of individuals belonging to same species. The definition in Encyclopaedia Britannica (https://www.britannica.com/science/population-ecology) is “A population is a subset of individuals of one species […]”. Therefore, a clarification (or alternative) is needed as the term is recurrently used across the manuscript.

Minor points:

- Ln 45 - The wording “…the antibiotic resistance…with the aim to…”. My belief is that the natural processes have NO aims but rather they are selected as beneficial or conferring an advantage to the organism. Therefore, I suggest rewording to get an appropriate scientific meaning.

- Lns 49-50 the sentence is quite imprecise. Please be more specific when referring to this “…increased bacterial antibiotic resistance”. Are you referring to pathogens? If so, please be specific.

- Ln 65 What exactly is the meaning of “low numbers”? Any counts of cell here?

- Not sure about Journal requirements but the norm is that the equipment, reagents and kits must be indicated with brand, CITY and country. The mentioning of city (all cases) and (in some cases) country are missing.

- Please mention the model of the photometer used to monitor the optical density

- Ln 135 – the abbreviation PMOR should be added here.

- Are these strains encoding single-copies of 16S rRNA genes too?

- Ln 137 – be always cautious using absolute terms like “perfect”. Science is not about perfection. “Specificity was evaluated as…good or high..” ore something similar.

- Ln 159 I suggest “resulted” instead of “was formed”

- Ln 173 space between “rep-”/ “and”

- If my main concern #1 is addressed, then additions to the Discussion and conclusions is expected.

- Figures/plots 2 and S2 have no explanations for the statistical analysis. What are the bars, how were they calculated? Materials and methods part is very scarce in explaining the statistical analysis.

**Reviewer 2**

**Recommendation: Major Revision**

Overview and general recommendation:

The development of antibiotic resistance at sub-MIC level or the “mutant selection window” hypothesis has been broadly studied and confirmed in vitro studies, and is crucial for undertanding the evolution of resistance in bacterial populations to optimize the dosage regimen. This paper focused the impact of selection pressure of ampicillin on a co-culture of two Thioalkalivibrio species, Tv. Thiocyanoxidans ARh2T and Tv. Versutus AL2T, by using rep- and qPCR to follow the cell growth dynamics of species over time at increasing concentrations of ampicillin, and mainly present a first-time descriptive study on growth competition between two new species. The paper is well-written and the content is on a topic of relevance and general interest to readers of the journal, but some of very important parts such as the aim of this study, experiment design and data interpretation are not clearly addressed or without statistic/quantitative analysis. Therefore, a major revision is warranted and my concerns are explained in more detail below.

Major comments:

Line55-62 Apart from the above paragraphs for introduction, author should address the main scientific problem more clearly, with focus on the significance of this study, instead of repeatly describing results here.

Line65-68 and Table1 Please explain why choose ampicillin and the logic underlying that species are exposed to dynamic concentrations in a single culture within four weeks, and the concentration range selected for testing. Besides, the test of sensitivity of pure cultures should be kept the same as the test of mix cultures (not just the concentration, but also the adaptation duration and testing methods) in order to compare the results. I suggest that it would be more clear to present the experimental design with a figure.

Line151-154 Instead of purely describing the growth behavior of each specie with different concentrations, it would be better to derivate the MIC value (perform quantitative analysis) to understand the sensitivity of pure cultures to ampicillin.

For discussion, apart from the “mutant selection window” hypothesis, author should centered on what they have found and discuss the science behind the results. Some of the discussion parts seem unrelated especially for line 184-211.

Minor comments:

Line 94 “ Error! Reference source not found” should be corrected.

Line 155 chemical analysis of ampicillin should be provided to understand actual exposure concentrations and degradation behavior during the experiment.

Fig 2 Please specify adapted culture1, 2 and 3.

**Author's Response to Decision Letter for (FEMSMC-2023-004)**

Dear Editor,

attached please find the revised version of the manuscript and the rebuttal to the comments of the reviewers. I hope that we addressed all comments well and that the manuscript will be accepted for publication.

With best regards,

Gerard Muyzer

Reviewer 1:

Comments to the Author

The manuscript (ID FEMSMC-2023-004) entitled ”Population dynamics of Thioalkalivibrio species under selective pressure of ampicillin” by Ahn et al. is a hypothesis-testing research dealing with a particular case of haloalkaliphilic community response to antibiotics.

I overall found the manuscript interesting and enjoyable to read. Authors used straightforward approaches to test antibiotic-driven changes in mixed cultures.

We would like to thank the reviewer for her/his kind words and for her/his time and efforts of reviewing our manuscript to improve it.

My concerns and suggestions are listed below:

1. The members of genus Thioalkalivibrio are now genome-sequences, therefore, I believe that addition of a short in silico analysis on the antibiotic determinants (eventually on those conferring beta-lactam resistance) would be of interest. I wonder whether the genome of AL2 strain encodes such genes. This analysis would be definitively prove insightful and may explain the experimental results.

We have added the in silico analysis on the genomes of both species. No specific beta-lactam resistance genes could be detected in either of the genomes, and only a limited set of genes were reported in the KEGG pathway map for beta-lactam resistance. However, multiple multidrug efflux pump, putative RND family efflux transporters, putative AcrAB-TolC efflux pumps and putative MFS transporters could be detected in both genomes with four genes specific to Tv. thiocyanoxidans ARh2T and eight to Tv. versutus AL2T. Amongst the supplementary efflux genes in Tv. versutus AL2T, one or multiple could be responsible for the increased resistance in Tv. versutus AL2T compared to Tv. thiocyanoxidans ARh2T, but transcriptomic and/or proteomic analysis would be needed to verify this. However, those analyses are outside the scope of this paper.

2. In the Abstract and Introduction, authors should briefly reasoning on why they experimented on this certain physiological (haloalkaliphiles) and metabolical (sulfur-oxidizers) types of bacteria.

Explanations were added to the abstract as well as the introduction to clarify the choice for working with Thioalkalivibrio.

3. In addition to the above concern, the authors are kindly asked to explain why they used ampicillin (beta-lactam antibiotic) that is usually effective against Gram-positives as the tested strains are Gram negative.

Ampicillin has the advantage of being a bactericidal antibiotic, but which only affects growing cells. This allows the bacteria to stay in a temporal growth arrest without being killed and to perform tolerance against the antibiotic (reduction of the biological cell processes and growth during a temporal bactericidal antibiotic exposure), and to develop possibly resistance by mutations. As a second reason for our choice of ampicillin is that we tested multiple antibiotics against Thioalkalivibrio strains and found that ampicillin was one of the most effective ones.

4. The use of the term “population” instead of “mixed culture” is problematic especially due to the established definition of biological population that consists of a number of individuals belonging to same species. The definition in Encyclopaedia Britannica (https://www.britannica.com/science/population-ecology) is “A population is a subset of individuals of one species […]”. Therefore, a clarification (or alternative) is needed as the term is recurrently used across the manuscript.

Thank you very much for this remark. We corrected the term “population” by a more appropriate vocabulary when needed throughout the manuscript.

Minor points:

- Ln 45 - The wording “…the antibiotic resistance…with the aim to…”. My belief is that the natural processes have NO aims but rather they are selected as beneficial or conferring an advantage to the organism. Therefore, I suggest rewording to get an appropriate scientific meaning.

Thank you very much for this correction, we adapted the sentence, that now reads: “However, antibiotic resistance as result of long-term evolution, existed long before anthropogenic pressure, protecting bacteria against their own antibiotic production and enabling them to outcompete other microbes in the ecosystem”

- Lns 49-50 the sentence is quite imprecise. Please be more specific when referring to this “…increased bacterial antibiotic resistance”. Are you referring to pathogens? If so, please be specific.

The sentence has been reformulated and reads now: “Applying sub-inhibitory concentrations in humans, livestock or the environment poses a major treat to bacterial disease treatments with antibiotics, as it leads to an increase of bacterial antibiotic resistance amongst pathogens.”

- Ln 65 What exactly is the meaning of “low numbers”? Any counts of cell here?

Unfortunately, no cell counts were made, only qPCR in which the cell number was found to be below the detection limit.

- Not sure about Journal requirements but the norm is that the equipment, reagents and kits must be indicated with brand, CITY and country. The mentioning of city (all cases) and (in some cases) country are missing.

We corrected this in the revised version of the manuscript.

- Please mention the model of the photometer used to monitor the optical density

The model of the photometer was added to the revised version of the manuscript.

- Ln 135 – the abbreviation PMOR should be added here.

The abbreviations are now added for a better clarity in brackets behind the gene name

- Are these strains encoding single-copies of 16S rRNA genes too?

Yes, both strains only possess one copy of the 16S rRNA gene. Thank you for this important remark, we added this information to the manuscript.

- Ln 137 – be always cautious using absolute terms like “perfect”. Science is not about perfection. “Specificity was evaluated as…good or high..” ore something similar.

The sentence has been reformulated and reads now: “The specificity of all three primers was evaluated as good after testing it in silico as well as by PCR using the genomic DNA of both species as well as a 1:1 mixture of DNA of both species (Figure S2).”

- Ln 159 I suggest “resulted” instead of “was formed”

The sentence has been reformulated and reads now: “The OD600 peak at the start of each Thioalkalivibrio culture resulted through the presence of elemental sulfur produced by the oxidation of thiosulfate”

- Ln 173 space between “rep-”/ “and”

A space was added. Thank you for mentioning it.

- If my main concern #1 is addressed, then additions to the Discussion and conclusions is expected.

We adjusted the results, discussion and conclusions sections accordingly.

- Figures/plots 2 and S2 have no explanations for the statistical analysis. What are the bars, how were they calculated? Materials and methods part is very scarce in explaining the statistical analysis.

Thank you for this helpful comment and we are sorry that it has escaped our attention. We added the needed information about the used statistical analysis to the figure captions as well as in the Material and Methods section.

Reviewer 2:

Comments to the Author

Overview and general recommendation:

The development of antibiotic resistance at sub-MIC level or the “mutant selection window” hypothesis has been broadly studied and confirmed in vitro studies and is crucial for undertanding the evolution of resistance in bacterial populations to optimize the dosage regimen. This paper focused the impact of selection pressure of ampicillin on a co-culture of two Thioalkalivibrio species, Tv. Thiocyanoxidans ARh2T and Tv. Versutus AL2T, by using rep- and qPCR to follow the cell growth dynamics of species over time at increasing concentrations of ampicillin, and mainly present a first-time descriptive study on growth competition between two new species. The paper is well-written and the content is on a topic of relevance and general interest to readers of the journal, but some of very important parts such as the aim of this study, experiment design and data interpretation are not clearly addressed or without statistic/quantitative analysis. Therefore, a major revision is warranted, and my concerns are explained in more detail below.

We would like to thank the reviewer for her/his critical review and her/his time invested to help us improving the manuscript.

Major comments:

Line 55-62 Apart from the above paragraphs for introduction, author should address the main scientific problem more clearly, with focus on the significance of this study, instead of repeatly describing results here.

We reformulated the paragraph and added information about the significance of the scientific problem. Moreover, we removed the results part from this paragraph. We hope that we could with this better address now the main scientific problem and the significance.

Line 65-68 and Table1 Please explain why choose ampicillin and the logic underlying that species are exposed to dynamic concentrations in a single culture within four weeks, and the concentration range selected for testing. Besides, the test of sensitivity of pure cultures should be kept the same as the test of mix cultures (not just the concentration, but also the adaptation duration and testing methods) in order to compare the results. I suggest that it would be more clear to present the experimental design with a figure.

Thank you very much for your question. Please find below the answers to your different questions:

(i) Choice of ampicillin: Ampicillin has the advantage of being a bactericidal antibiotic, but which only affects growing cells. This allows the bacteria to stay in a temporal growth arrest without being killed and to perform tolerance against the antibiotic (reduction of the biological cell processes and growth during a temporal bactericidal antibiotic exposure), and to develop possibly resistance by mutations. As a second reason for our choice of ampicillin is that we tested multiple antibiotics against Thioalkalivibrio strains and found that ampicillin was one of the most effective ones.

(ii) Exposure to dynamic concentrations for four weeks: Regarding the logic underlying of the dynamic concentration, the approach of stepwise increasing the antibiotic concentration with every transfer cycle allows resistance and mutations to emerge and has previously been applied (example: van der Horst, et al., 2011; doi: 10.1089/mdr.2010.0101). The concentration of 0.1 µg/ml was chosen as starting concentration as it was the highest concentration on which both strains were able to grow. The duration range of 4 weeks is linked to the fact that after that time, no improvement in increasing the antibiotic concentration could have been made meaning that the culture reached its higher resistance concentration (The cultures were never able to grow at 25.6 µg/ml of ampicillin).

(iii) Sensitivity test of pure cultures: We apologize. As we presented the adaptation phases in the article, it was not clear how we proceeded. We transferred the adapting cultures every 2-3 days to stay below the 4 days, after which ampicillin was degraded. At each step, we tried to transfer our culture to medium with the double of ampicillin concentration, but we also transferred the same culture again to the same ampicillin concentration with fresh antibiotic and medium. This was in case the culture would not directly grow at the increased antibiotic concentration and needed more time to adapt at the lower concentration. Then again, after 2-3 days we tried to transfer our culture to the double concentration of ampicillin until no growth could be obtained anymore after multiple trials (which happened at 25.6 µg/ml). Therefore, the same number of days between the transfers was applied as were tested in the sensitivity test of the pure cultures. We added a Figure (Figure S1) visualizing the process. We reformulated our text to make our experimental design clearer and Table 1 (it changed now to supplementary Table S1 as more information was added and it became too big) accordingly. We also adapted our numeration of the experimental timeline (before we counted as time when the culture started, but it is more logic to take the time when the sample was taken from the culture), which is include in Table S1 and was adapted for Figure 2. Thank you very much for your question and helping us to pinpoint unclarities.

Line151-154 Instead of purely describing the growth behavior of each specie with different concentrations, it would be better to derivate the MIC value (perform quantitative analysis) to understand the sensitivity of pure cultures to ampicillin.

Thank you very much for your comment. We added the information about the MIC value in the manuscript.

For discussion, apart from the “mutant selection window” hypothesis, author should centered on what they have found and discuss the science behind the results. Some of the discussion parts seem unrelated especially for line 184-211.

Thank you for your comment, we integrated and linked this paragraph better to our results.

Minor comments:

Line 94 “Error! Reference source not found” should be corrected.

Thank you very much for notifying us, we removed all links, so that this error is not occurring anymore.

Line 155 chemical analysis of ampicillin should be provided to understand actual exposure concentrations and degradation behavior during the experiment.

We would have liked to perform this analysis; however, appropriate samples were not taken during the experiment to allow measuring the ampicillin concentration.

Fig 2 Please specify adapted culture1, 2 and 3.

The adapted cultures 1, 2 and 3 are triplicates from the same initial culture, which were kept all time separately from the beginning. We added this information to the figures caption.

**Reviews of manuscript FEMSMC-2023-004.r1**

**Reviewer 1: Horia Banciu, Babes-Bolyai University**

**Recommendation: Minor Revision**

Minor corrections:

- Ln 58 – ‘major threat’ instead of ‘major treat’

- at first sight, the differences between figures S4 and S5 depicting the detected antibiotic resistance genes in genomes of the two Thioalkalvibrio species are barely visible despite differences outlined in the Results part. I suggest re-checking of these figures and, eventually, highlighting the genese that are specific to one or another strain.

**Reviewer 2**

**Recommendation: Minor Revision**

The manuscript presented the dynamics of co-cultured two species under the growing pressure of antibiotic ampicillin. It basically explained each part clearly and provided a full picture of what researchers have done. I have no more questions apart from that only in silico analysis of ampicillin resistance genes was performed. It would be better to provide more genetic/sequencing data on how two species responded to ampicillin perturbation. Before accepted, some misspelling need to be corrected.

**Author's Response to Decision Letter for (FEMSMC-2023-004.r1)**

Dear Editor,

Attached please find the rebuttal and the corrected manuscript.

We hope that the manuscript is now suited for acceptance.

With best regards,

Gerard Muyzer

Reviewer 1:

Comments to the Author

Minor corrections:

- Ln 58 – ‘major threat’ instead of ‘major treat’

We would like to thank the reviewer for noticing this misspelling. We corrected it and scanned the entire document again for misspellings.

- at first sight, the differences between figures S4 and S5 depicting the detected antibiotic resistance genes in genomes of the two Thioalkalvibrio species are barely visible despite differences outlined in the Results part. I suggest re-checking of these figures and, eventually, highlighting the genes that are specific to one or another strain.

Yes, you are correct, there are no visual differences in the KEGG pathway map of encoded beta-lactam resistance genes for both strains. For genes taken into account in the KEGG pathway map, only one RND family efflux transporter, MFP subunit (B0684_RS10505) was listed supplementarily in Tv. versutus AL2T compared to Tv. thiocyanoxidans ARh2T. This information was included in the previous manuscript (Please see Ln 199-202 in the final manuscript) and is also represent in Table S2 (We marked by “X” the genes included in the KEGG pathway map and it can be seen that there is no counter representative for this gene in Tv. thiocyanoxidans ARh2T).

Reviewer 2:

Comments to the Author

The manuscript presented the dynamics of co-cultured two species under the growing pressure of antibiotic ampicillin. It basically explained each part clearly and provided a full picture of what researchers have done. I have no more questions apart from that only in silico analysis of ampicillin resistance genes was performed. It would be better to provide more genetic/sequencing data on how two species responded to ampicillin perturbation.

We would like to thank the reviewer for her/his kind words. We agree with the reviewer that there appear to be no major differences in the 'gene repertoire' between both species. It is probable that distinctions may emerge at the transcriptomic and proteomic levels, which could provide a more comprehensive explanation for the differences in ampicillin sensitivity observed in both species. However, unfortunately, conducting such analyses falls beyond the scope of this paper. Our primary focus here was to examine the dynamics of the two species under ampicillin-induced stress.

Before accepted, some misspelling need to be corrected.

Thank you for notifying us. We scanned and corrected again the entire manuscript for misspellings.

**Editor’s comments for manuscript FEMSMC-2023-004.r2**

Thank you for submitting the above manuscript to our journal. It has now been expertly refereed, and they found the work to be sound. However I have a few changes that need to be made before final acceptance for publication.

The Results section headings should not be the based on methods or be the same as in the materials and methods section. Instead they should reflect the results. The following headings need to be revised to reflect the results not the method.

- Line 153 - heading needs to be revised to one that reflect the results not methods.

- Line 164 - heading needs to be revised to one that reflect the results.

- Line 199 - The heading is identical to the one in the methods section.

For clarity, all acronyms / abbreviations should be written in full when first mentions. Please write out quantitative PCR (qPCR) and repetitive-element PCR (rep-PCR) in the text when first mentioned. They should also be fully written in the headings.

**Author's Response to Decision Letter for (FEMSMC-2023-004.r2)**

Dear Editor,

We have made the changes as requested and hope that the manuscript is now good enough to be accepted for publication.

with best regards,

Gerard Muyzer
